# A predictor model of treatment resistance in schizophrenia using data from electronic health records

**DOI:** 10.1371/journal.pone.0274864

**Published:** 2022-09-19

**Authors:** Giouliana Kadra-Scalzo, Daniela Fonseca de Freitas, Deborah Agbedjro, Emma Francis, Isobel Ridler, Megan Pritchard, Hitesh Shetty, Aviv Segev, Cecilia Casetta, Sophie E. Smart, Anna Morris, Johnny Downs, Søren Rahn Christensen, Nikolaj Bak, Bruce J. Kinon, Daniel Stahl, Richard D. Hayes, James H. MacCabe

**Affiliations:** 1 Institute of Psychiatry, Psychology and Neuroscience, King’s College London, London, United Kingdom; 2 Department of Psychiatry, University of Oxford, Oxford, United Kingdom; 3 South London and Maudsley NHS Foundation Trust, London, United Kingdom; 4 Shalvata Mental Health Center, Hod Hasharon, Israel; 5 Sackler Faculty of Medicine, Tel Aviv University, Tel Aviv, Israel; 6 MRC Centre for Neuropsychiatric Genetics and Genomics, Cardiff University, Cardiff, United Kingdom; 7 H. Lundbeck A/S, Copenhagen, Denmark; 8 Lundbeck Pharmaceuticals LLC, Deerfield, IL, United States of America; Fukuoka University, JAPAN

## Abstract

**Objectives:**

To develop a prognostic tool of treatment resistant schizophrenia (TRS) in a large and diverse clinical cohort, with comprehensive coverage of patients using mental health services in four London boroughs.

**Methods:**

We used the Least Absolute Shrinkage and Selection Operator (LASSO) for time-to-event data, to develop a risk prediction model from the first antipsychotic prescription to the development of TRS, using data from electronic health records.

**Results:**

We reviewed the clinical records of 1,515 patients with a schizophrenia spectrum disorder and observed that 253 (17%) developed TRS. The Cox LASSO survival model produced an internally validated Harrel’s C index of 0.60. A Kaplan-Meier curve indicated that the hazard of developing TRS remained constant over the observation period. Predictors of TRS were: having more inpatient days in the three months before and after the first antipsychotic, more community face-to-face clinical contact in the three months before the first antipsychotic, minor cognitive problems, and younger age at the time of the first antipsychotic.

**Conclusions:**

Routinely collected information, readily available at the start of treatment, gives some indication of TRS but is unlikely to be adequate alone. These results provide further evidence that earlier onset is a risk factor for TRS.

## 1. Introduction

Approximately 20–34% of patients with schizophrenia continue to experience persistent symptoms despite adequate treatment with at least two antipsychotic drugs, and are termed treatment resistant [1, 2]. Treatment-resistance in schizophrenia (TRS) is associated with up to 11 times greater direct healthcare costs [3] and a delay in detection of TRS is associated with worse outcomes such as longer hospitalisation and delay in symptomatic improvement [4–6]. Therefore, early identification of TRS patients is a key priority [7], and modelling has shown that even modest predictive power may have clinical utility [8].

At present there is some evidence which indicates that TRS patients show abnormalities in glutamate transmission, comparatively normal dopamine transmission, and significant decreases in grey matter compared to treatment-responsive patients [9]. However, these abnormalities can only be detected using expensive and invasive brain scans, and have only modest predictive validity [10]. Genetic tests would be cheaper, but also lack predictive power [9]. In contrast, clinical and demographic information from electronic health records (EHRs) is readily available, non-invasive and inexpensive. A small number of previous studies have used clinical information to predict TRS. A recent systematic literature review of observational research [11] identified young age at illness onset as a robust predictor of TRS. In addition, other factors that were associated with TRS were psychiatric diagnosis, level of functioning, male gender, and season of birth. However, existing observational research has suffered from three main limitations. Firstly, studies using population registries have lacked detailed clinical information [12, 13]. Secondly, studies that have investigated incident cases of psychosis longitudinally have been subject to significant attrition [1], therefore potentially not capturing service users who are most unwell. Thirdly, most studies have adopted traditional frequentist statistical approaches that are not optimised for prediction [11].

This study aimed to develop a prognostic tool of TRS in a large and diverse clinical cohort, with comprehensive coverage of patients using mental health services in a defined geographical area. To achieve this, we approximated as closely as possible the operationalized TRRIP [14] definition of TRS (which is a consensus guidelines that operationalise criteria for determining TRS, providing a benchmark for research, in view of previous inconsistencies in defining and measuring treatment resistance and response) and examined a range of possible predictors using clinically rich data from EHRs.

## 2. Material and methods

### 2.1. Setting

South London and Maudsley (SLAM) NHS Foundation Trust serves a geographic catchment of 1.36 million residents across four London boroughs (Lambeth, Southwark, Lewisham and Croydon). EHRs have been used by SLAM in all its services since 2006. In 2008, the Clinical Record Interactive Search (CRIS) system was developed [15, 16], to allow researchers to search and retrieve anonymised SLAM EHRs, with over 450,000 cases currently represented in the system. The anonymisation process has been previously described in detail [15].

CRIS derives detailed information from both structured (i.e. drop-down menu) and free-text (i.e. clinical progress notes) fields from EHRs. The extraction of free-text information is facilitated by Natural Language Processing (NLP) applications [15, 16], which consider the linguistic context within which words appear, therefore offering a more sophisticated approach to basic key word searches [16]. Where more complex judgements are required, anonymised text can be viewed by a researcher and manually coded.

### 2.2. Ethics statement

Ethical approval as an anonymised database for secondary analysis by the Oxford C Research Ethics Committee (18/SC/0372) [15, 16]. The database operates on opt-out basis.

### 2.3. Cohort

Using structured and free-text data in CRIS, all patients who had a diagnosis of schizophrenia, schizotypal or delusional disorder (ICD-10 codes F20-F29) between 1^st^ January 2007 and 31^st^ December 2017; were prescribed antipsychotics in this period; and lived within the SLAM catchment area, or were homeless but receiving treatment from SLAM services, at the time of the prescription of their first antipsychotic medication, were identified.

### 2.4. Treatment resistant schizophrenia definition

A structured field for TRS was not available, and it was not possible to develop an NLP application that could parse the complex sequence of decisions required to arrive at a TRS diagnosis. Therefore, to determine the point at which a patient became TRS, we developed a bespoke algorithm to target potentially relevant EHRs, which were then manually examined. TRS was defined as evidence of failure to respond to two different antipsychotic prescriptions for six or more weeks, and a prescription of a third new antipsychotic. A six-week antipsychotic trial duration was chosen as a pre-requisite following NICE guidelines [17] and TRRIP TRS definition [14]. A patient was also classified as TRS, if one of their three antipsychotics was clozapine. In the UK almost all prescriptions of clozapine are for TRS- The British National Formulary, which provides the official national prescribing guidelines, lists only two indications for clozapine (1) Schizophrenia in patients unresponsive to, or intolerant of, conventional antipsychotic drugs; (2) Psychosis in Parkinson’s disease. As our cohort did not include patients with the latter diagnosis we assumed that cases of clozapine was likely to be an indicator of TRS.

**Fig 1:**
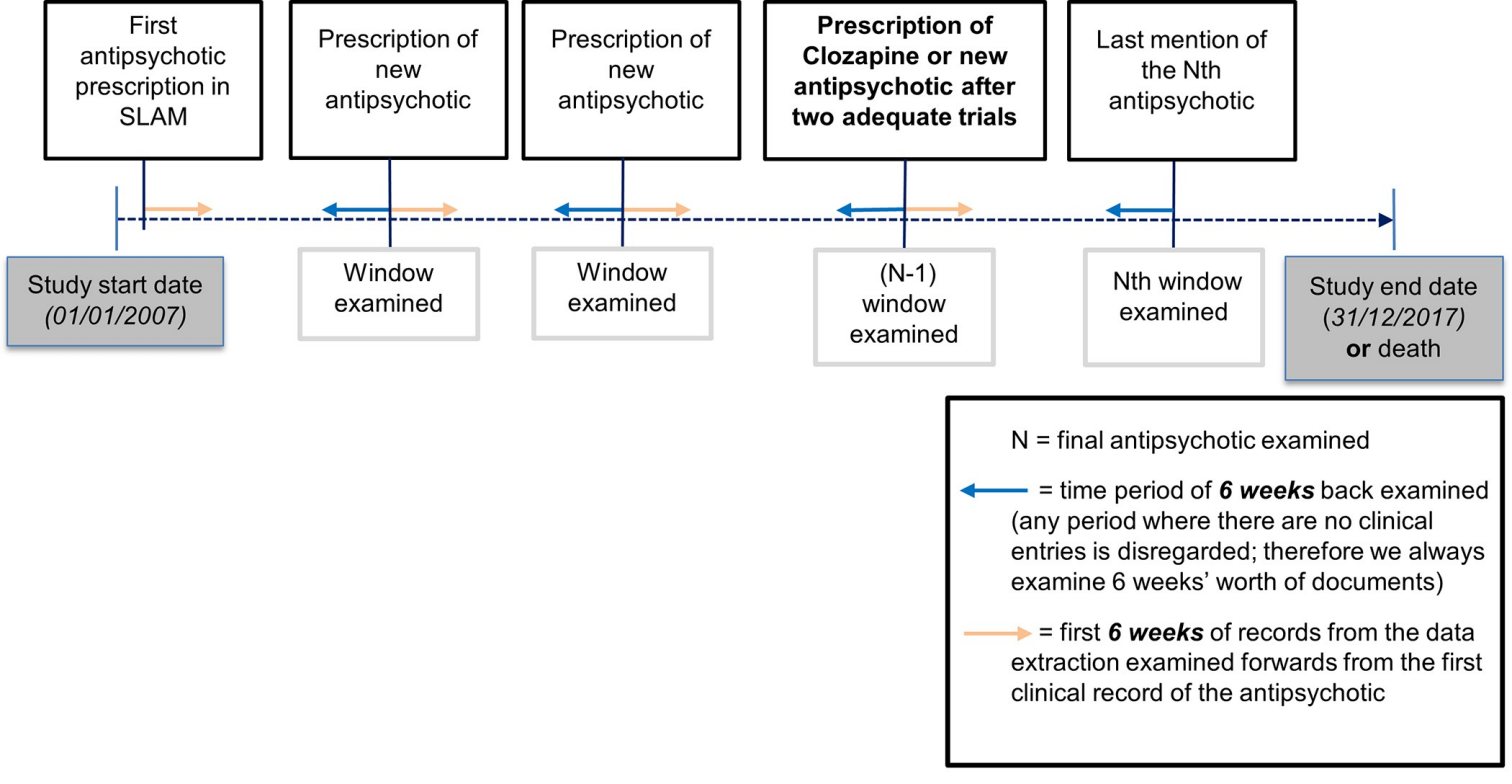
Antipsychotic coding process timeline for coding outcome data.

Fig 1 illustrates the process of manually coding the antipsychotic treatment trials. All antipsychotic trials prescribed since 1/01/2007 were examined to determine if they were: 1) an adequate trial, lasting for at least six weeks (we assessed this by examining the first six weeks of EHRs following an antipsychotic prescription) and 2) a qualifying trial, meaning that antipsychotics were switched due to failure to respond as opposed to treatment non-adherence or treatment side-effects. To determine this we examined the EHRs for the six weeks before and after a medication switch. Where the reasons were unclear, if no evidence of switching medication due to non-adherence or side-effects was detected, then treatment failure was assumed to be the reason. For each patient, this process was repeated for all identified antipsychotic trials until TRS was identified or there were no further antipsychotics prescribed. S1 Fig summarises the decision-making process to identifying TRS.

Since manually coding case-notes was very time-consuming, taking approximately one hour per patient, it was not feasible to manually code all patients who met the inclusion criteria to determine their TRS status. Consequently, a random sample comprising 10% of all patients who met the above inclusion criteria was selected for manual coding. S2 Fig describes this process. Where the EHRs indicated that fewer than three antipsychotics were prescribed, the patient was coded as not treatment resistant (unless there was a prescription of clozapine). EHRs of patients where there was evidence of three or more antipsychotics being prescribed were examined in detail following the procedures outlined above.

### 2.5. Extraction of other data from CRIS

Data regarding all antipsychotic drugs listed in the British National Formulary (BNF) 65 [18] were extracted. Medication data were derived from structured fields, SLAM pharmacy records and free-text fields. Medication data from the free-text were extracted using General Architecture for Text Engineering (GATE) software [19], a suite of tools that facilitates the use of NLP applications and features. The NLP application used to extract antipsychotic medication has been validated previously [16, 20, 21]. Specific filters were applied to maximize precision and recall on instances of antipsychotic prescribing. For example, all instances of medication prescription that did not include a dose value were excluded at the point of data extraction.

Socio-demographic, socioeconomic, clinical and service use information was extracted within 3 months of the first antipsychotic date (also termed index date). Socio-demographic and socioeconomic variables included age, gender, ethnicity, and area-level deprivation index. Seventeen ethnic group categories were collapsed into “White” (including: British, Irish and other White Backgrounds), “Black” (including: African, Caribbean, White and Black African, White and Black Caribbean and any other Black background) and “Other” (including: Bangladeshi, Chinese, Indian, Pakistani, White and Asian, any other Asian background, any other mixed background, any other ethnic group or ethnicity not stated), due to small numbers in these categories. We used an area-level index of multiple deprivation, linked to the patients’ post-code, as a proxy for socioeconomic status ascertained from 2007 UK Census [22]. In this case, multiple deprivation indices were applied to lower super output areas (LSOAs), which are the smallest enumeration unit, each containing on average 1,500 residents [22]. In addition, homelessness [23] was ascertained based on ‘no fixed abode’ codes.

Clinical factors included primary and comorbid ICD-10 diagnoses and Health of the Nation Outcome Scale (HoNOS). Primary diagnosis was assigned in a hierarchical manner as schizoaffective (F25-25.9), schizophrenia (F20-F20.9) or other prolonged psychosis (F28-F29.9) after consulting with the clinical team. This approach was adopted in order to deal with multiple changes in diagnosis over time. Comorbid diagnoses included personality disorder (F60-61.9), substance use (F10-14.9, F16-16.9, F18-19.9), developmental disabilities (F70-79.9; F80-84.9; F90-90.9), anxiety disorders (F40-41.9; F42- 43.9; F43.1), and mood disorders (F30-31.9; F32-33.9; F34.0–34.1; F42.1; F34.8–39.9). Clinical symptom presence and severity was estimated from HoNOS, which is a clinical outcome instrument, composed of 12 items designed to measure behaviour, impairment, symptoms, and social functioning [24]. HoNOS information was retrieved in the following hierarchical fashion: i) closest but before the index date (within 3 months), if no HoNOS was identified we then searched for ii) HoNOS closest but up to three months after index date, and iii) any HoNOS before the index date. Items are scored on a five-point scale. Due to small cell sizes, subscale scores were collapsed into three categories: 0 “no problem”; 1 “minor problem requiring no action”; 2–4 ‘‘significant problem” [25].

Service use was ascertained by: the number of days where there were face-to-face contacts with outpatient services; number of days spent as an in-patient; patients were also classified into three categories depending on whether they were hospitalised at the time of the first antipsychotic prescription post 2007: not in hospital; compulsory admission under Mental Health Act 1983; or voluntary admission.

### 2.6. Statistical analyses

The analyses were done in R using the following packages: glmnet [26], caret [27], pROC [28], StatMatch [29], and c060 [30]. Model development, validation and presentation were conducted according to the guidelines outlined by Steyerberg et al [31] and to the Transparent Reporting of a multivariable prediction model for Individual Prognosis Or Diagnosis (TRIPOD) statement [32]. A survival analysis was conducted with TRS as the outcome. The date of the first antipsychotic prescribed on or after 1^st^ January 2007 was considered time zero. The end of the individual follow-up period was TRS date (the date on which the patient was switched to a third new antipsychotic due to non-response and/or was prescribed clozapine), date of death or 31^st^ December 2017, which ever occurred first.

The decision to use a statistical-learning method was determined by two factors: the need to maximise predictive power and minimise overfitting. Therefore, the Least Absolute Shrinkage and Selection Operator (Lasso [33])- a linear method automatically performing variable selection and more specifically the Lasso version of Cox regression [33] was chosen. We assessed whether our sample size was sufficient for a Cox model including our number of candidate predictor parameters, with our rate and mean follow-up years according to Riley and colleagues [34]. Missing data in the exposures were imputed with *k*-nearest neighbours (KNN [35]) imputation using the Gower distance [36]. In the model building for regularised regression the tuning parameter lambda for the Lasso models was estimated with 100-time repeated 10-fold cross-validation (CV). Tuning was done by minimising the partial likelihood deviance. The one-standard error (SE) rule model, which provides a better compromise between reliable variable selection and good prediction accuracy [37], was selected.

The model’s discriminative performance was evaluated with Harrell’s concordance index (C-index [38]). For an assessment of calibration, the calibration slope for Cox-Lasso model was returned [31]. Measures of positive predictive value (PPV; i.e. precision), negative predictive value (NPV), sensitivity and specificity at 1, 2, 5 and 10 years were estimated for the survival model using the Youden index [39] maximising the sum of sensitivity and specificity (giving the same importance to false positive and false negative errors) and the PPV was compared with the prevalence at each time point using a ratio (*δ* = PPV/prevalence). All measures of performance were internally validated using 100-time repeated 10-fold cross-validation optimism-correction as for Harrell [40].

The Lasso Cox model is presented as a *nomogram* [31]. The nomogram presents prognostic indexes (PI) [41], which are related to the corresponding survival probabilities at 1, 2, 5 and 10 years for ease of interpretation. Kaplan-Meier curves of the unadjusted TRS survival probabilities for levels of the selected variables are presented. Additionally, sensitivity analyses were performed to assess the robustness of the survival model prediction accuracy by excluding: 1) patients whose first antipsychotic date was in the first 3 months of 2007, in order to account for the possibility that this date was not a true reflection of when they began their antipsychotic treatment; 2) patients who died during the observation period.

## 3. Results

In total 15,129 patients met the inclusion criteria and the information for 1,515 patients was manually coded (a random 10% of the total cohort). Out of the patients who were manually coded, 627 were identified as having three or more antipsychotic medication prescriptions. Out of those cases, 206 (33%) were identified as TRS, 413 (66%) non-TRS, and 7 (1%) were not categorised due to lack of information. Data checking identified inconsistencies (observed in 5.2%), leading to changes in the TRS date of 1.4% of all manually coded cases, namely changing their TRS date. Out of the 888 patients with fewer than three antipsychotics recorded, 47 (5%) were prescribed clozapine and therefore categorised as TRS. The remaining patients were categorised as non-TRS (N = 841, 95%). Overall, 253 (17%) patients were identified as having TRS, 1,255 (83%) as non-TRS, and 7 (0.5%) did not have sufficient information to be categorised and were not included in the analysis.

The analysis included data from 1435 individuals between 01/01/2007 and 31/12/2017 (see S2 Fig). The number of potential predictors in the model was 31. For a Cox model with 181 events (we excluded 72 patients due to being prescribed clozapine at the start of the observation window, and one who died few days afterwards, see S2 Fig) at, 8946.4 person-years, mean follow-up time of 6.23 years, 47 parameters in the model and a Cox-Snell adjusted R-squared of 0.30 or larger, a sample size of 1430 is sufficient to minimise overfitting and to ensure precise estimates of key parameters [34]. Median time to event was 2232 days (interquartile range = 1075–3694, range 2–4016). The period prevalence of TRS was 12.6% (95% CI: 10.9–14.3): 181 TRS and 1254 non-TRS.

**Fig 2:**
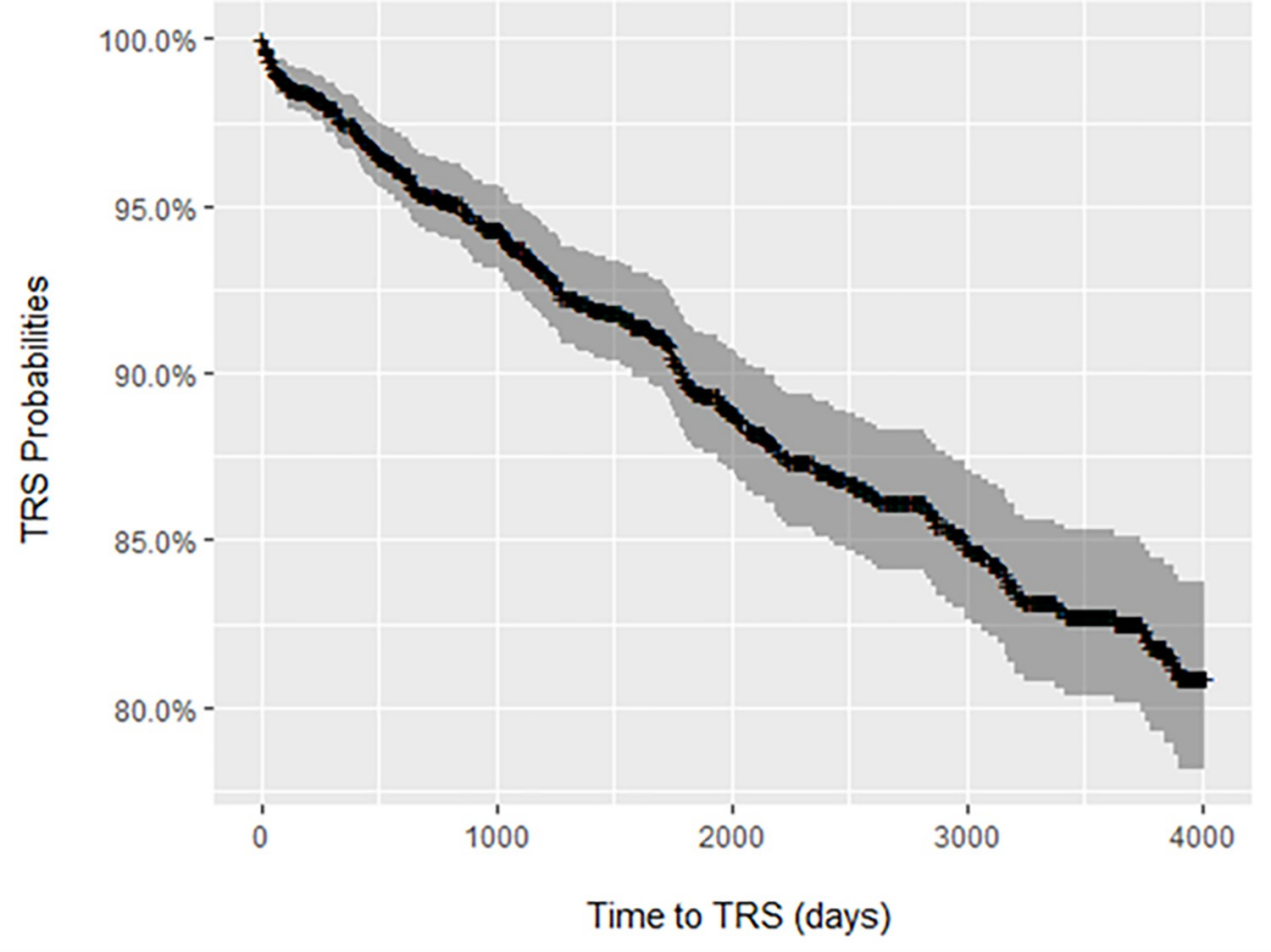
Kaplan-Meier curve of the survival probability for treatment resistant schizophrenia.

Table 1 summarises the distribution of the exposure variables for the overall cohort and for those patients who were identified as TRS. The data contained 1044 (72.75%) complete cases, and the mean percentage of missing data per variable was 9.52% (SD 9.84, range 0–21.88%). Fig 2 is a Kaplan-Meier curve of the survival probability of TRS, which illustrates that the hazard of developing TRS was approximately constant across time in the study time window (1^st^ January 2007- 31^st^ December 2017).

**Table 1:** Descriptive statistics of treatment resistant schizophrenia predictors.

Exposures	Overall (n = 1435)	TRS (n = 181, 12.6%)	Missing data
*Continuous*	*Median (25* ^*th*^ *-75* ^*th*^ *p)*	*Median (25* ^*th*^ *-75* ^*th*^ *p) Count (% within TRS)*	Count overall (%), Count TRS (% within count overall)
*Categorical*	*Count (% overall)*		
***Socio-demographics and Socioeconomics***			
Age (years)	40.1 (29.5–51.9)	37.6 (28.3–46.6)	0
Gender			
Male (R)	807 (56.2)	110 (60.8)	0
Female	628 (43.76)	71 (39.2)	
Ethnicity			
Black (R)	611 (42.6)	95 (52.5)	18 (1.3), 0
White	569 (39.7)	62 (34.3)	
Other	237 (16.5)	24 (13.3)	
Deprivation score	31.9 (24.5–37.7)	32.1 (25.9–38.5)	18 (1.3), 0
***Main diagnosis***			
Schizophrenia (R)	526 (36.7)	72 (39.8)	0
Schizoaffective	52 (3.6)	4 (2.2)	
Other prolonged psychosis	351 (24.5)	19 (10.5)	
Undetermined	506 (35.3)	86 (47.5)	
***Comorbidities***			
Personality			
Yes	61 (4.3)	8 (4.4)	0
No (R)	1374 (95.7)	173 (95.6)	
Any substance use			
Yes	94 (6.6)	11 (6.1)	0
No (R)	1341 (93.4)	170 (93.9)	
Developmental disabilities			
Yes	63 (4.4)	9 (5)	0
No (R)	1372 (95.6)	172 (95)	
Anxiety related disorders			
Yes	83 (5.8)	7 (3.9)	0
No (R)	1352 (94.2)	174 (96.1)	
Mood disorders			
Yes	283 (19.7)	21 (11.6)	0
No (R)	1152 (80.3)	160 (88.4)	
***Service use***			
Inpatient/on MHA section:			
Not inpatient (R)	1063 (74.1)	134 (74)	0
Compulsory inpatient	271 (18.9)	33 (18.2)	
Voluntary inpatient	101 (7)	14 (7.7)	
Community face-to-face days pre- index	2 (1–4)	3 (1–5)	0
Community face-to-face days post index	4 (1–9)	5 (2–10)	0
Inpatient days pre-index	0 (0–1)	0 (0–1)	0
Inpatient days post index	0 (0–15.50)	0 (0–23)	0
Symptoms: HoNOS			
1. Overactive, agitated behaviour			
No problem (R)	569 (39.7)	76 (42)	268 (18.7), 7(10.1)
Minor problem, no action	268 (18.7)	32 (17.7)	
Significant problem	330 (23)	46 (25.4)	
2. Non- accidental self-injury			
No problem (R)	981 (68.4)	135 (74.6)	271 (18.9), 28(10.3)
Minor problem, no action	91 (6.3)	10 (5.5)	
Significant problem	92 (6.4)	8 (4.4)	
3. Drinking or drug-taking			
No problem (R)	794 (55.3)	109 (60.2)	289 (20.1), 30(10.4)
Minor problem, no action	119 (8.3)	13 (7.2)	
Significant problem	233 (16.2)	29 (16)	
4. Cognitive problems			
No problem (R)	555 (38.7)	78 (43.1)	274 (19.1), 28 (10.2)
Minor problem, no action	285 (19.9)	45 (24.9)	
Significant problem	243 (16.9)	30 (16.6)	
5. Physical illness or disability			
No problem (R)	715 (49.8)	100 (55.2)	278 (19.4), 29 (10.4)
Minor problem, no action	193 (13.4)	21 (11.6)	
Significant problem	249 (17.4)	31 (17.1)	
6. Hallucinations and delusions			
No problem (R)	316 (22)	44 (24.3)	275 (19.2), 27 (9.8)
Minor problem, no action	131 (9.1)	18 (9.9)	
Significant problem	713 (49.7)	92 (50.8)	
7. Depressed mood			
No problem (R)	447 (31.1)	66 (36.5)	275 (19.2), 28 (10.2)
Minor problem, no action	325 (22.6)	44 (24.3)	
Significant problem	275 (19.2)	43 (23.8)	
8. Other mental and behavioural problems			
No problem (R)	309 (21.5)	46 (25.4)	279 (19.4), 29 (10.4)
Minor problem, no action	205 (14.3)	35 (19.3)	
Significant problem	642 (44.7)	71 (39.2)	
9. Relationship problems			
No problem (R)	428 (29.8)	62 (34.3)	281 (19.6), 30 (10.4)
Minor problem, no action	275 (19.2)	30 (16.6)	
Significant problem	451 (31.4)	59 (32.6)	
10. Activities of daily living			
No problem (R)	543 (37.8)	68 (37.6)	286 (19.9), 28 (9.8)
Minor problem, no action	284 (19.8)	37 (20.4)	
Significant problem	322 (22.4)	48 (26.5)	
11. Living conditions			
No problem (R)	645 (44.9)	78 (43.1)	314 (21.9), 33 (10.5)
Minor problem, no action	229 (16)	37 (20.4)	
Significant problem	247 (17.2)	33 (18.2)	
12. Occupation and activities			
No problem (R)	463 (32.3)	61 (33.7)	310 (21.6), 34 (11.0)
Minor problem, no action	257 (17.9)	29 (16)	
Significant problem	405 (28.2)	57 (31.5)	
8.a HoNOS Other mental and behavioural problems–Type			
Phobic, anxiety, obsessive-compulsive			
Yes	299 (20.8)	39 (21.5)	268 (18.7), 27 (10.1)
No (R)	868 (60.5)	115 (63.5)	
Mental strain/tension			
Yes	259 (18)	28 (15.5)	268 (18.7), 27 (10.1)
No (R)	908 (63.3)	126 (69.6)	
Dissociative, somatoform			
Yes	24 (1.7)	5 (2.8)	268 (18.7), 27 (10.1)
No (R)	1143 (79.7)	149 (82.3)	
Eating, sleep, sexual			
Yes	284 (19.8)	39 (21.5)	268 (18.7), 27 (10.1)
No (R)	883 (61.5)	115 (63.5)	

Abbreviations: p = percentile; R = reference category; TRS = treatment resistant schizophrenia; PTSD = post-traumatic stress disorder; PICU = Psychiatric Intensive Care Unit; EIS = Early intervention team; MHA = Mental Health Act, HoNOS = Health of the Nation Outcome Scales.

S1 Table summarises the model performance. The internally validated estimate of C-index for Lasso-Cox regression was 0.60, indicating a weak prediction model. The validated calibration slope of 1.27 indicated that the Cox model was slightly underfitting and therefore underestimating risk for patients at high risk while overestimating for patients at low risk. The validated ratio *δ* between PPV and prevalence was always greater than one, reaching 1.69 at 2 years. S2 Table summarises the validated estimates of the model’s performance- for example at 2 years the model achieved a validated PPV 0.04 (prevalence at 2 years was 0.02); NPV 0.98; sensitivity 0.60; specificity 0.63.

Table 2 displays estimated Lasso coefficients for all 31 potential predictors (47 main effect parameters). Seven variables were selected as predictors of TRS in the Lasso-Cox model (corresponding to the 1 SE penalty): having more inpatient days in the three months before and after the first antipsychotic; having more community face-to-face clinical contact in the three months before the first antipsychotic; having a minor cognitive problem requiring no action (vs not having a cognitive problem or having a severe one) according to HoNOS score, and younger age at the time of the first antipsychotic. Having a diagnosis of other prolonged psychosis emerged as a protective factor to developing TRS. The Kaplan-Meier survival curves per levels of the identified predictors are presented in S3–S10 Figs.

**Table 2:** Survival analysis Lasso Cox regression selected predictors (one standard error penalty).

Predictor of TRS	Lasso Cox	
n = 1435	Log HR	Effect dir.
Diagnosis = ‘other prolonged psychosis’‘	-0.301	-
Inpatient days post index	0.001	+
Community face-to-face clinical contacts (1/day) pre index	0.010	+
Inpatient days pre-index	0.007	+
HoNOS Cognitive (‘minor problem, no action’)	0.089	+
Age	-0.010	-

Abbreviations: SCZ = schizophrenia; PTSD = post-traumatic stress disorder; PICU = Psychiatric Intensive Care Unit; EIS = Early intervention team; MHA = Mental Health Act, HoNOS = Health of the Nation Outcome Scales, OMP = Other mental and behavioural problems, inc. = increase, rel. = relative, dir. = direction.

**Fig 3:**
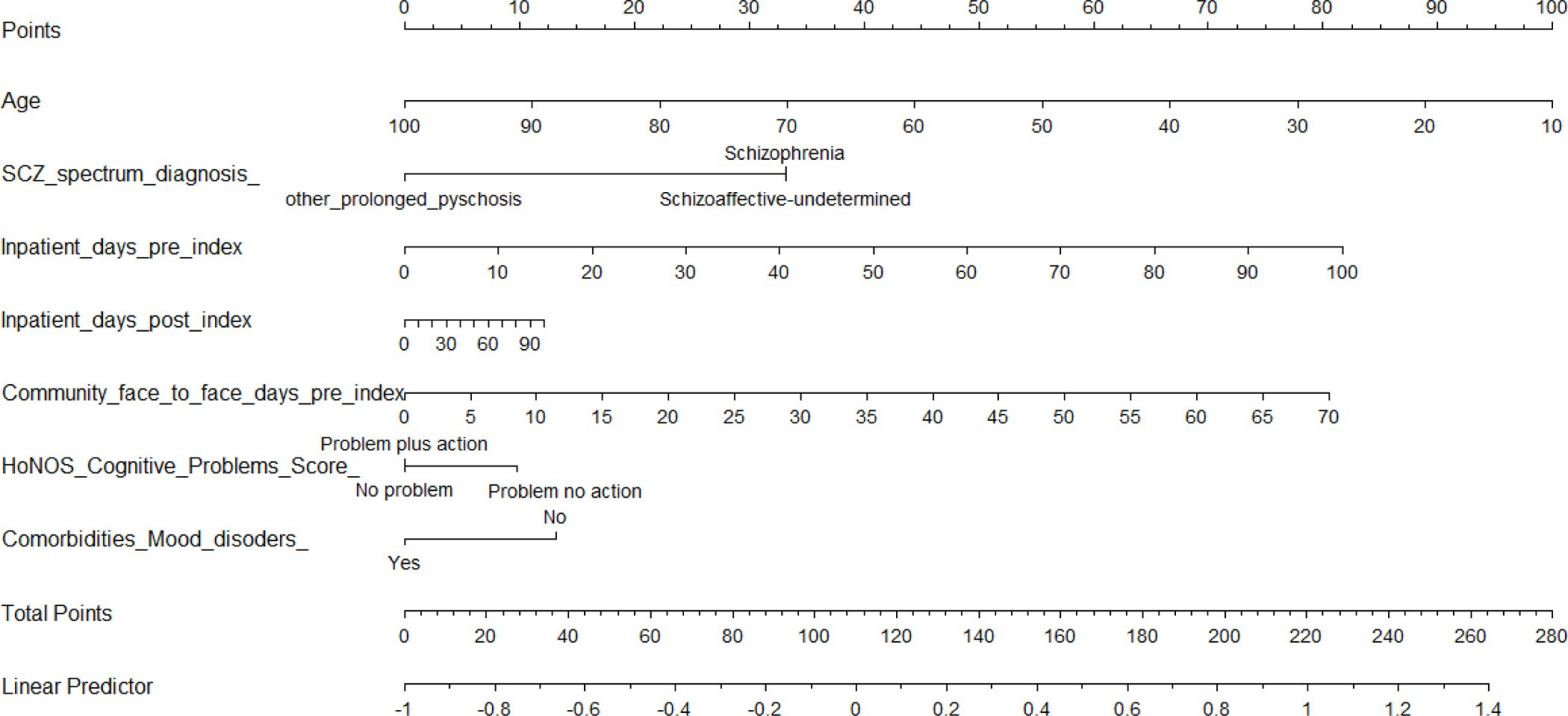
Nomogram for Cox Lasso regression to calculate individual normalized prognostic indexes (PI, given by the linear predictor line) for treatment resistant schizophrenia. Coefficients are based on the Lasso Cox model.—To predict the rate of survival using the nomogram, one can take the example of a 30-year-old patient, with Schizophrenia, having 34 inpatient days recorded at 3 months before the 1st antipsychotic (AP) date, 50 inpatient days recorded 3 months after 1st AP date, 15 community face-to-face days recorded 3 months before 1st AP date, having a minor problem requiring no action and having mood disorders as comorbidity, has a total point score of 78 + 33 + 28 + 6 + 18 + 10 + 0 = 173. This corresponds to a normalized prognostic index of 0.57 (linear predictor line) for TRS, meaning that the patient has a probability to become TRS at 1 year falling in the range 4.14%-7.66%, at 2 years falling in the range 7.62%-13.89%, at 5 years in the range 16.45%-28.74% and at 10 years in the range 26.57%-44.13% (see S3 Table).

Fig 3 is a nomogram for Cox Lasso regression to calculate individual normalized prognostic indexes, given by the linear predictor line, for TRS. The coefficients are based on the Lasso Cox model. The nomogram allows us to compute the normalized prognostic index (PI) for a new patient. The PI is a single-number summary of the combined effects of a patient’s risk factors and is a common method of describing the risk for an individual. In other words, the PI is a linear combination of the risk factors, with the estimated regression coefficients as weights. The exponentiated PI gives the relative risk of each patient in comparison with a baseline patient. The PI is normalized by subtracting the mean PI. S3 Table further describes the normalized prognostic indexes (PI) for TRS translated into probabilities of developing TRS at 1, 2, 5 and 10 years. Appendix 1 describes how to compute the PI in further detail.

In total 361 patients were already taking antipsychotics at the time the EHRs database was established. These were included in the main analysis, but their date of first antipsychotic may have been before 1 Jan 2007. Therefore, we conducted a sensitivity analysis where we excluded patients with the first antipsychotic date within the first 3 months of 2007 (n = 361 excluded, see S4 Table). We also performed a sensitivity analysis excluding patients who died in the observation window (n = 168 excluded) (see S5 Table). Both sensitivity models performed less well than the full model in relation to validated discrimination and calibration, but the corrected C-indexes were similar across all three analyses, which supported the robustness of the full model and further suggests that the events of death and TRS are not competing risks (see S6 Table).

## 4. Discussion

Our results indicated that the best predictors of TRS were having a diagnosis of schizophrenia or schizoaffective disorder (as opposed to a diagnosis of other psychosis); more inpatient days (in the three months before and after the first antipsychotic); having more community face-to-face clinical contact (in the three months before the first antipsychotic); having a minor cognitive problem requiring no action (as opposed to no problem or having a severe one); and younger age at the time of the first antipsychotic.

The prevalence of TRS was 17%, which is somewhat lower than previous studies in this field [1, 12, 42, 43]. One possible explanation is the use of more stringent criteria for TRS than some previous studies. In line with existing NICE guidelines [17], our study pre-specified that an adequate trial had a minimum of six weeks, as opposed to other previous research, which has allowed trials of shorter duration [1]. Having a shorter antipsychotic trial would translate to patients being classified as TRS earlier in their treatment trajectory. Furthermore, while many studies have used proxies such as hospital admissions [12] to determine response to each antipsychotic, we manually rated response to ensure that each switch in antipsychotic medication was due to non-response as opposed to side-effects or non-adherence. This represents a more robust way of determining TRS, which is in line with TRRIP guidance [14].

Our results further indicated that the hazard of developing TRS was constant over a period of 10 years. This suggests that the probability of developing TRS does not stabilise over the course of patients’ treatment trajectory. This is consistent with the findings of Wimberley et al [12], which also reported that the cumulative incidence of TRS (assessed by different indicators including 90-day polypharmacy), increased steadily over time, but does not seem to fit with data showing that the majority of TRS patients are treatment resistant from the outset [1, 7]. However, both the current study and the Wimberley et al [12] study defined TRS from switches in antipsychotic initiated by clinicians, which in turn rely on clinicians recognising, and acting on, treatment failure. There is good evidence that the detection of TRS in clinical services is often delayed by months or years [44]. Thus, the data are compatible with a situation in which TRS develops early in many patients, but is only recognised by clinicians, and thus identified by our algorithm, months or years later.

With a C-statistic of 0.60, the prediction accuracy of the final model was not sufficient to be of clinical use alone. However, in line with existing literature [12, 13, 45, 46], we found that socio-demographic, clinical and service use predictors of TRS were younger age at the illness onset, a diagnosis of other prolonged psychosis, and higher levels of service use, as measured by inpatient days and clinical face-to-face contacts. The identified predictors were robust and remained in sensitivity analyses, where we excluded patients whose prescription of the first antipsychotic was in the first three months of the observation window (which are more likely to be patients with prolonged psychosis); and patients who died between 01/01/2007 and 31/12/2017.

Younger age at onset is the most consistent risk factor identified to date for TRS [11], and this study provides further evidence in support of this. The presence of minor cognitive problems needing no action (an item on the HoNOS scale) was also associated with TRS. Other research has identified verbal intelligence and fluency as associated with TRS [47]. The fact that mild, but not more severe cognitive impairment was identified is consistent with clinical impressions that the level of cognitive impairment associated with schizophrenia is mild and generally does not require specific intervention. However, it is important to highlight here that what we report in this study is the patient’s and clinician’s perception of cognitive problems, as captured by HoNOS, as opposed to cognitive performance captured by standardised cognitive testing.

This study had several strengths. SLAM is one of Europe’s largest mental health providers and is near-universal provider of mental health care in its geographical area. Therefore, we were able to capture a large and representative sample of patients who receive national health care provision in this area. In addition, CRIS is a longitudinal data source, which encompasses clinical information from both structured and free-text fields from both inpatient and outpatient clinical settings. This enabled us to investigate TRS over a ten-year period and examine rich and diverse clinical information and therefore a wide range of possible predictors. Furthermore, our analyses were conducted on a manually coded dataset, which not only identified antipsychotic episodes in accordance with existing clinical guidelines [14], but was also able to determine reasons for switching, something that has been problematic in previous research.

There are several potential limitations that need to be considered when interpreting our findings. It is possible that our definition of TRS, which required both failed trials of antipsychotics to exceed six weeks duration, could have led to underestimating TRS cases. Less than six weeks may be adequate in some cases to establish non-response, and such trials were not counted in our algorithm. On the other hand, employing a stricter criterion to establishing TRS arguably ensures that fewer false positive cases were detected. In addition, we did not consider antipsychotic dose in assessing the adequacy of the antipsychotic trials and further research could explore the role of this on the time it takes for a patient to become TRS. The present study only considered medication trials from 01/01/2007 onwards. Consequently, some individuals may have become TRS earlier than the date identified. In relation to psychiatric diagnosis of the service users in this cohort, we included individuals who had schizophrenia, schizoaffective and other prolonged psychosis diagnosis due to the low number of patients diagnosed with schizophrenia at their illness onset, which is likely to reflect a reluctance to diagnose people with schizophrenia early in their illness. Furthermore, the prescription of the first antipsychotic from 01/01/2007 was not necessarily the patients’ first treatment, we minimised this by discounting all patients who appeared to have their first antipsychotic prescription in the first three months of the database (01/01/07–30/03/07) in the sensitivity analyses. We were only able to include factors, which were recorded in the clinical notes and had sufficient information present to allow us to include in the models. Therefore, we were unable to include potential predictors, such as smoking, due to large number of missing data. Lastly, it is important to note that inpatient days/clinical events/clinical contacts may not be constant throughout treatment (there may be periods of higher contact, namely at illness onset and around TRS date). Therefore, if a patient became TRS close to the start of the observation period (01/01/2007), this patient might be receiving frequent contact over the limited follow-up period and would appear to be a more frequent user of services than someone who became TRS later.

Our findings have potential implications for further clinical research. With a C-statistic of 0.60, our model has insufficient predictive power to be clinically useful in isolation, but it is possible that the risk factors identified here could be incorporated into a multivariate model including information from biomarkers, such as neuroimaging or genetic markers [10]. Moreover, the internally validated PPV for the survival model predicting TRS at each time point was larger than the prevalence at the same time point. Therefore, if one were to use this model as a stratification tool to identify patients with a higher chance of developing TRS, for example when recruiting patients for an RCT, fewer patients would be needed to yield a similar amount of TRS patients. For example, a trial recruiting patients identified as having a high probability of TRS by the model will yield 1.69 times as many TRS patients in 2 years than a trial recruiting unselected patients. It will therefore need to recruit 41% fewer patients to obtain a given number of TRS patients at 2 years. In relation to further research, the external validity of the model examined here can be further tested by replicating the study on a similar cohort from other settings. In conclusion, routinely collected information, readily available at the start of treatment, gives some indication of TRS but is unlikely to be adequate alone. Future studies that develop clinical risk prediction tools can combine these predictors with other identified predictors, which will increase the likelihood of success. In addition, further work investigating the biological underpinnings would be extremely useful.

## Supporting information

S1 Table Lasso Cox performance. The optimism-corrected performance was obtained via 100-time repeated 10-fold cross-validation. C-index = Harrell’s concordance statistic.
(DOCX)

S2 Table Validated performances at 1, 2, 3, 4, 5 and 10 years. (DOCX)

S3 Table Normalized prognostic indexes (PI) for treatment resistant schizophrenia (TRS) translated into probabilties of developing TRS at 1, 2, 5 and 10 years. (DOCX)

S4 Table Lasso Cox regression (one standard error penalty) selected predictors excluding patients whose first antipsychotic date was in the first 3 months of 2007 (n = 1074). (DOCX)

S5 Table Lasso Cox regression (one standard error penalty) selected predictors excluding patients who died in the window (n = 1267). (DOCX)

S6 Table Performance for Lasso Cox for the sensitivity analyses (the optimism-corrected performance was obtained via 100-time repeated 10-fold cross-validation). (DOCX)

S1 Fig Treatment resistant schizophrenia decision-making process for coding outcome data. (DOCX)

S2 Fig Cohort identification flow-chart. (DOCX)

S3 Fig Kaplan-Meier curve of the survival probabilities for treatment resistant schizophrenia (TRS), differentiating patients who were predicted to develop TRS and those who were predicted not to. (DOCX)

S4 Fig Kaplan-Meier curve of the survival probabilities for TRS by diagnosis. (DOCX)

S5 Fig Kaplan-Meier curve of the survival probabilities for treatment resistant schizophrenia (TRS) by the severity of cognitive problems (HoNOS assessment). (DOCX)

S6 Fig Kaplan-Meier curve of the survival probabilities for treatment resistant schizophrenia (TRS) by the presence of a mood disorder. (DOCX)

S7 Fig Kaplan-Meier curve of the survival probabilities for treatment resistant schizophrenia (TRS) by age (median split of 40 years). (DOCX)

S8 Fig Kaplan-Meier curve of the survival probabilities for treatment resistant schizophrenia (TRS) by number of inpatient days before the prescription of the first antipsychotic from 01/01/2007 (median split of 4 inpatient days). (DOCX)

S9 Fig Kaplan-Meier curve of the survival probabilities for treatment resistant schizophrenia (TRS) by number of inpatient days after the prescription ossf the first antipsychotic from 01/01/2007 (median split of 14 inpatient days). (DOCX)

S10 Fig Kaplan-Meier curve of the survival probabilities for treatment resistant schizophrenia (TRS) by number of face-to-face clinical contacts in the three months before the prescription of the first antipsychotic from 01/01/2007 (median split of 3 clinical contacts). (DOCX)

S1 Appendix Computing the prognostic index. (DOCX)
