# Supplementary material for: A predictor model of treatment resistance in schizophrenia using data from electronic health records
Source: PLoS One. 2022 Sep 19;17(9):e0274864. doi: 10.1371/journal.pone.0274864 (PMC9484642; doi:10.1371/journal.pone.0274864)
Supplement: S1 Table — The optimism-corrected performance was obtained via 100-time repeated 10-fold cross-validation. C-index = Harrell’s concordance statistic. (DOCX) [file pone.0274864.s001.docx]

**Supplementary Table 1.** **Lasso Cox performance.** *The optimism-corrected performance was obtained via 100-time repeated 10-fold cross-validation. C-index=Harrell’s concordance statistic.*

| **Measure** | **Lasso Cox regression** | |
| --- | --- | --- |
|  | **Apparent** | **Corrected** |
| C-index | 0.66 | 0.60 |
| Calibration slope | 1.80 | 1.27 |
| PPV/prevalence (1 year) | 2.07 | 1.47 |
| PPV/prevalence (2 years) | 2.08 | 1.69 |
| PPV/prevalence (5 years) | 1.48 | 1.12 |
| PPV/prevalence (10 years) | 1.53 | 1.29 |
