# Supplementary material for: A predictor model of treatment resistance in schizophrenia using data from electronic health records
Source: PLoS One. 2022 Sep 19;17(9):e0274864. doi: 10.1371/journal.pone.0274864 (PMC9484642; doi:10.1371/journal.pone.0274864)
Supplement: S2 Table — (DOCX) [file pone.0274864.s002.docx]

**Supplementary Table 2. Validated performances at 1, 2, 3, 4, 5 and 10 years**

|  | **1 year** | **2 years** | **3 years** | **4 years** | **5 years** | **10 years** |
| --- | --- | --- | --- | --- | --- | --- |
| **AUC** | 0.74 | 0.71 | 0.67 | 0.68 | 0.65 | 0.64 |
| **PPV** | 0.03 | 0.04 | 0.04 | 0.06 | 0.08 | 0.16 |
| **NPV** | 0.99 | 0.98 | 0.98 | 0.98 | 0.96 | 0.92 |
| **SEN** | 0.66 | 0.60 | 0.64 | 0.63 | 0.58 | 0.60 |
| **SPE** | 0.62 | 0.63 | 0.56 | 0.59 | 0.60 | 0.59 |
| **Prevalence** | 0.01 | 0.02 | 0.03 | 0.04 | 0.06 | 0.12 |
