# Supplementary material for: A predictor model of treatment resistance in schizophrenia using data from electronic health records
Source: PLoS One. 2022 Sep 19;17(9):e0274864. doi: 10.1371/journal.pone.0274864 (PMC9484642; doi:10.1371/journal.pone.0274864)
Supplement: S3 Table — (DOCX) [file pone.0274864.s003.docx]

**Supplementary Table 3. Normalized prognostic indexes (PI) for treatment resistant schizophrenia (TRS) translated into probabilties of developing TRS at 1, 2, 5 and 10 years.**

| Quantile | Normalized PI for TRS | Probability (%) of TRS at 1 year | Probability (%) of TRS at 2 years | Probability (%) of TRS at 5 years | Probability (%) of TRS at 10 years |
| --- | --- | --- | --- | --- | --- |
| 0% | -0.72 | 1.20 | 2.24 | 5.01 | 8.45 |
| 10% | -0.31 | 1.81 | 3.37 | 7.47 | 12.50 |
| 20% | -0.19 | 2.04 | 3.80 | 8.39 | 14.00 |
| 30% | -0.11 | 2.21 | 4.12 | 9.08 | 15.09 |
| 40% | -0.04 | 2.35 | 4.37 | 9.62 | 15.96 |
| 50% | 0.01 | 2.48 | 4.61 | 10.14 | 16.78 |
| 60% | 0.06 | 2.60 | 4.83 | 10.61 | 17.53 |
| 70% | 0.10 | 2.73 | 5.05 | 11.08 | 18.28 |
| 80% | 0.17 | 2.90 | 5.38 | 11.77 | 19.36 |
| 90% | 0.26 | 3.16 | 5.85 | 12.77 | 20.92 |
| 97.5% | 0.53 | 4.14 | 7.62 | 16.45 | 26.57 |
| 100% | 1.16 | 7.66 | 13.89 | 28.74 | 44.13 |
