# Supplementary material for: A predictor model of treatment resistance in schizophrenia using data from electronic health records
Source: PLoS One. 2022 Sep 19;17(9):e0274864. doi: 10.1371/journal.pone.0274864 (PMC9484642; doi:10.1371/journal.pone.0274864)
Supplement: S4 Table — (DOCX) [file pone.0274864.s004.docx]

**Supplementary Table 4: Lasso Cox regression (one standard error penalty) selected predictors excluding patients whose first antipsychotic date was in the first 3 months of 2007 (n=1074).**

| **Predictor of TRS** | **Lasso Cox** | |
| --- | --- | --- |
|  | Log HR | Effect  direction |
| SCZ spectrum diagnosis (OPP vs SCZ) | -0.236 | - |
| Age | -0.008 | - |
| Ethnicity (‘White’ vs ‘Black’) | -0.066 | - |
| Inpatient days post index | 0.003 | + |
| HoNOS OMBP (‘significant problem’) | -0.023 | - |
| HoNOS Cognitive (‘minor problem, no action’) | 0.044 | + |
| Community face-to-face clinical contacts (1/day) pre index | 0.008 | + |

*Abbreviations: AP=antipsychotic, SCZ=schizophrenia; PTSD=post-traumatic stress disorder; PICU= Psychiatric Intensive Care Unit; EIS=Early intervention team; MHA=Mental Health Act, HoNOS=Health of the Nation Outcome Scales, OMBP=Other mental and behavioural problems, inc.=increase, rel.=relative, OPP=Other prolonged psychosis*.
