# Supplementary material for: A predictor model of treatment resistance in schizophrenia using data from electronic health records
Source: PLoS One. 2022 Sep 19;17(9):e0274864. doi: 10.1371/journal.pone.0274864 (PMC9484642; doi:10.1371/journal.pone.0274864)
Supplement: S5 Table — (DOCX) [file pone.0274864.s005.docx]

**Supplementary Table 5: Lasso Cox regression (one standard error penalty) selected predictors excluding patients who died in the window (n=1267).**

| **Predictor of TRS** | **Lasso Cox** | |
| --- | --- | --- |
|  | Log HR | Effect direction |
| SCZ spectrum diagnosis other prolonged psychosis (vs ‘SCZ’) | -0.327 | - |
| Comorbidities Mood disorders (‘yes’) | -0.153 | - |
| Inpatient days post index | 0.003 | + |
| Inpatient days pre index | 0.002 | + |
| HoNOS Cognitive Problems (‘minor problem, no action’) | 0.175 | + |
| HoNOS OMBP (‘minor problem, no action’) | 0.002 | + |
| HoNOS Dissociative Somatoform (‘yes’) | 0.225 | + |
| SCZ spectrum diagnosis undetermined | 0.009 | + |
| HoNOS Act. Daily Living Problems (‘minor problem, no action’) | 0.036 | + |
| HoNOS OMBP (‘significant problem’) | -0.042 | - |
| Community face-to-face clinical contacts (1/day) pre index | 0.013 | + |

*Abbreviations: SCZ=schizophrenia; PTSD=post-traumatic stress disorder; PICU= Psychiatric Intensive Care Unit; EIS=Early intervention team; MHA=Mental Health Act, HoNOS=Health of the Nation Outcome Scales, OMBP=Other mental and behavioural problems, inc.=increase, rel.=relative*
