# Supplementary material for: A predictor model of treatment resistance in schizophrenia using data from electronic health records
Source: PLoS One. 2022 Sep 19;17(9):e0274864. doi: 10.1371/journal.pone.0274864 (PMC9484642; doi:10.1371/journal.pone.0274864)
Supplement: S6 Table — (DOCX) [file pone.0274864.s006.docx]

**Supplementary Table 6. Performance for Lasso Cox for the sensitivity analyses (the optimism-corrected performance was obtained via 100-time repeated 10-fold cross-validation).**

| **Measure** | **Lasso Cox regression** | |
| --- | --- | --- |
|  | **Apparent** | **Corrected** |
| **C-index** |  |  |
| 1^st^ AP (n=1074) | 0.66 | 0.57 |
| Deaths (n=1267) | 0.66 | 0.58 |
| **Calibration slope** |  |  |
| 1^st^ AP (n=1074) | 3.06 | 4.41 |
| Deaths (n=1267) | 1.75 | 0.62 |

*Abbreviations: C-index= Harrell’s concordance statistic, 1^st^ AP=sensitivity analysis 1 excluding patients with the first antipsychotic date within the first 3 months of 2007, Deaths=sensitivity analysis 2 excluding patients who died in the observation window.*
