# Supplementary material for: A predictor model of treatment resistance in schizophrenia using data from electronic health records
Source: PLoS One. 2022 Sep 19;17(9):e0274864. doi: 10.1371/journal.pone.0274864 (PMC9484642; doi:10.1371/journal.pone.0274864)
Supplement: S1 Fig — (DOCX) [file pone.0274864.s007.docx]

**Supplementary Figure 1:** **Treatment resistant schizophrenia decision-making process for coding outcome data**


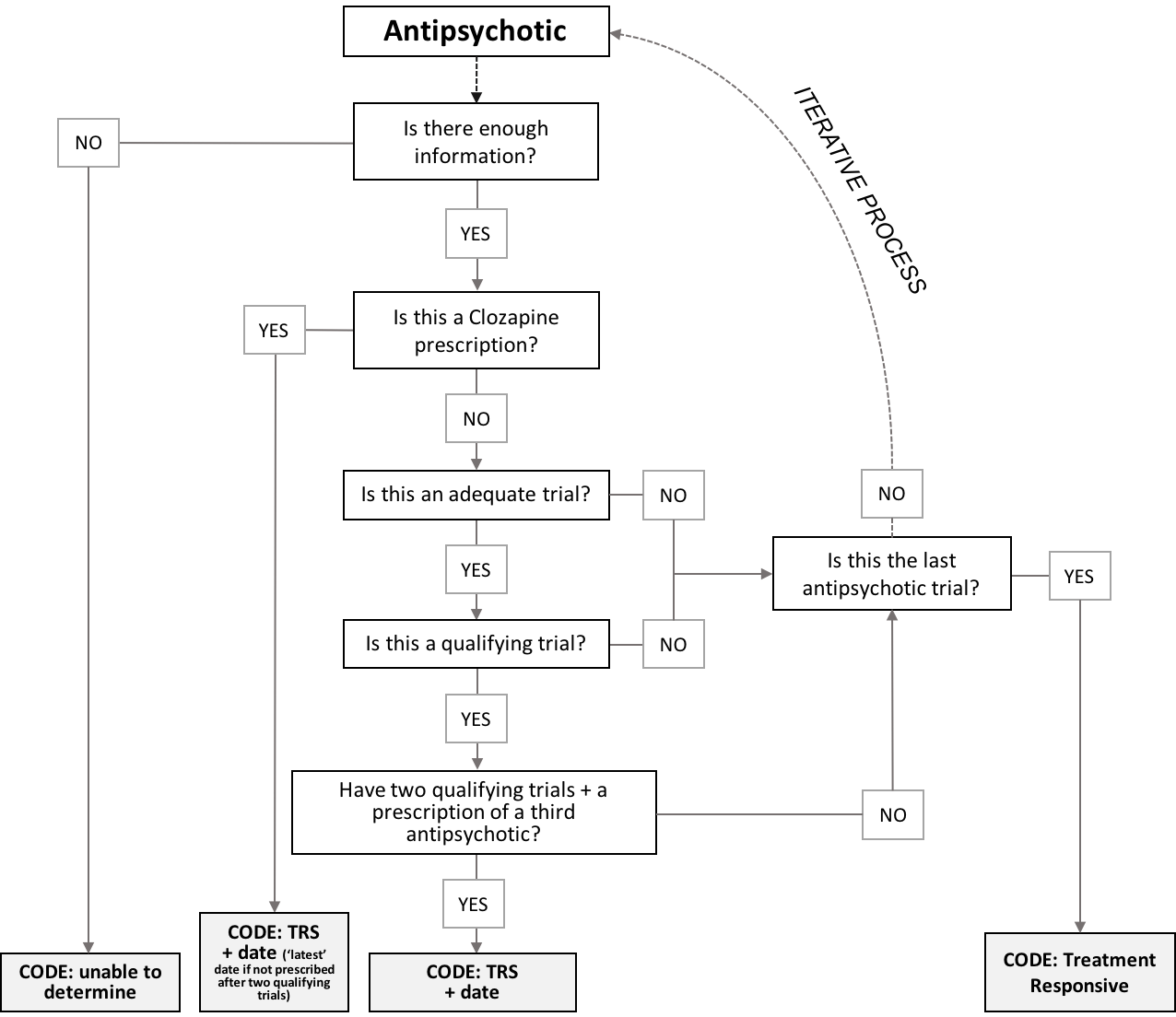


*
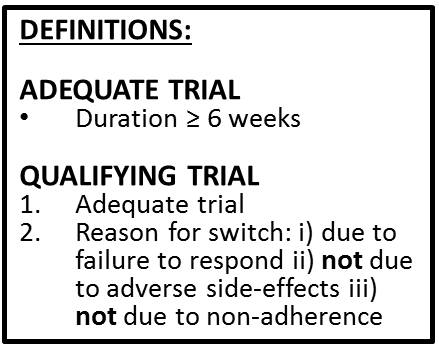
*
