# Supplementary material for: A predictor model of treatment resistance in schizophrenia using data from electronic health records
Source: PLoS One. 2022 Sep 19;17(9):e0274864. doi: 10.1371/journal.pone.0274864 (PMC9484642; doi:10.1371/journal.pone.0274864)
Supplement: S2 Fig — (DOCX) [file pone.0274864.s008.docx]

**Supplementary Figure 2: Cohort identification flow-chart**

≥ 3 antipsychotics

N=6,248

(41%)

10% randomly selected and coded

(TRS only when prescribed clozapine)

N=888

10% randomly selected and coded manually

N=627

≤ 2 antipsychotics

N=8,881

(59%)

Total number of SLAM patients with records in the CRIS dataset at 31/12/2017

(N=330,030)

Patients without a diagnosis of a non-affective psychotic disorder

(N=302,479)

Patients excluded (N=12,422)

- No mention of antipsychotics between 2007 and 2017 (n=6,861)
- No evidence of residence within SLaM catchment area or homelessness at first from 01/01/2007 antipsychotic mention (n=5,561)

Number of patients with a recorded diagnosed with a non-mood psychotic disorder at the date (F20-F29)

(N=27,551)

Number of patients fulfilling inclusion criteria

(N=15,129)

TRS: 206 (33%)

Non-TRS: 413 (66%)

Missing: 7 (1%)

TRS: 47 (5%)

Non-TRS: 841 (95%)

TRS: 253 (17%)

Non-TRS: 1255 (83%)

Missing: 7 (0.05%)

Data extraction done for 1515 cases

Data extraction

(N=1515)

Excluded:

- 71 patients had TRS at study start (were prescribed clozapine)
- 1 patient died on 3/1/2007 with index date 18/01/2007
- 8 patients had addresses outside the catchment area

1435

Patients included
