# Supplementary material for: A predictor model of treatment resistance in schizophrenia using data from electronic health records
Source: PLoS One. 2022 Sep 19;17(9):e0274864. doi: 10.1371/journal.pone.0274864 (PMC9484642; doi:10.1371/journal.pone.0274864)
Supplement: S3 Fig — (DOCX) [file pone.0274864.s009.docx]

**Supplementary Figure 3: Kaplan-Meier curve of the survival probabilities for treatment resistant schizophrenia (TRS), differentiating patients who were predicted to develop TRS and those who were predicted not to.**

**
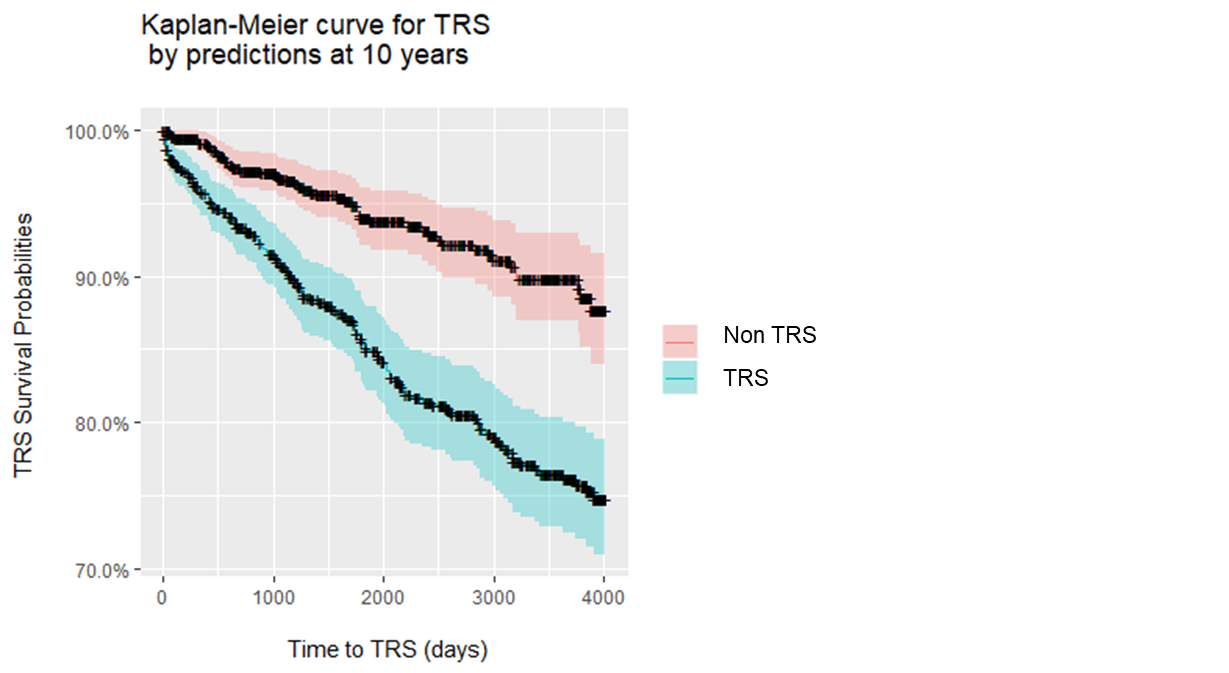
**
