# Supplementary material for: A predictor model of treatment resistance in schizophrenia using data from electronic health records
Source: PLoS One. 2022 Sep 19;17(9):e0274864. doi: 10.1371/journal.pone.0274864 (PMC9484642; doi:10.1371/journal.pone.0274864)

**Supplementary Figure 5: Kaplan-Meier curve of the survival probabilities for treatment resistant schizophrenia (TRS) by the severity of cognitive problems (HoNOS assessment)**


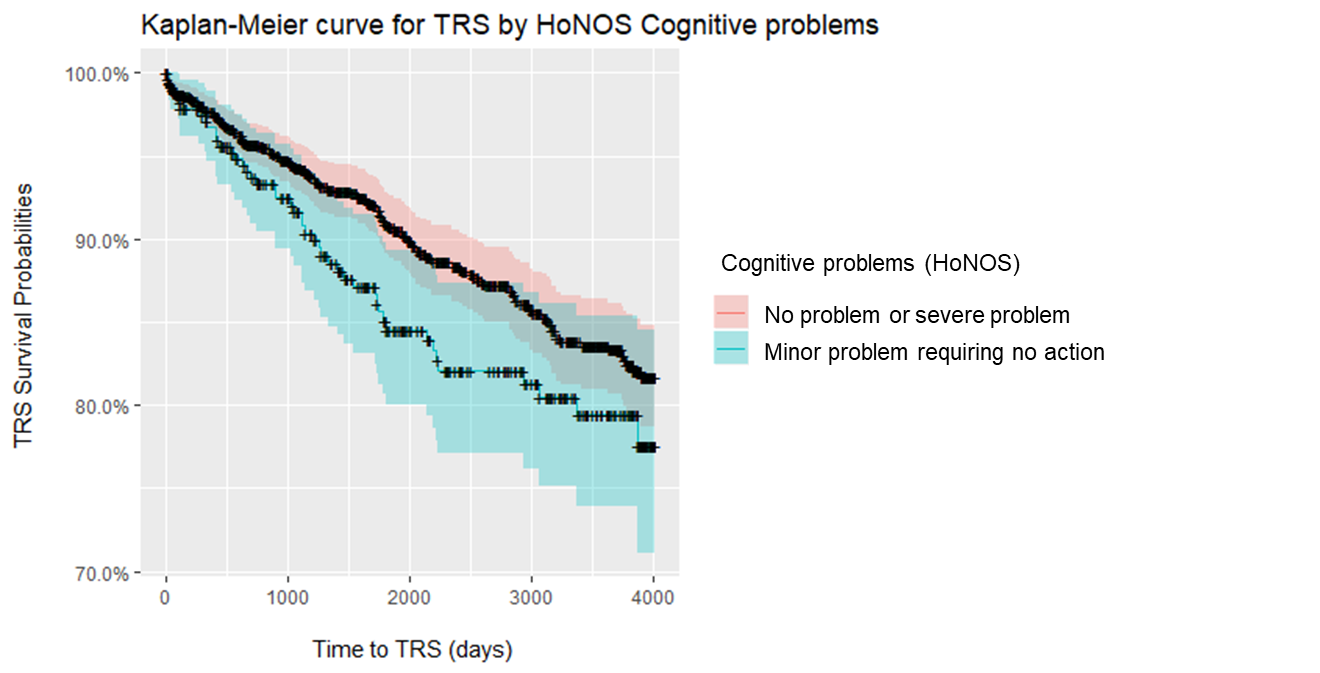

Supplement: S5 Fig — (DOCX) [file pone.0274864.s011.docx]
