# Supplementary material for: A predictor model of treatment resistance in schizophrenia using data from electronic health records
Source: PLoS One. 2022 Sep 19;17(9):e0274864. doi: 10.1371/journal.pone.0274864 (PMC9484642; doi:10.1371/journal.pone.0274864)

**Supplementary Figure 7**: **Kaplan-Meier curve of the survival probabilities for treatment resistant schizophrenia (TRS) by age (median split of 40 years)**


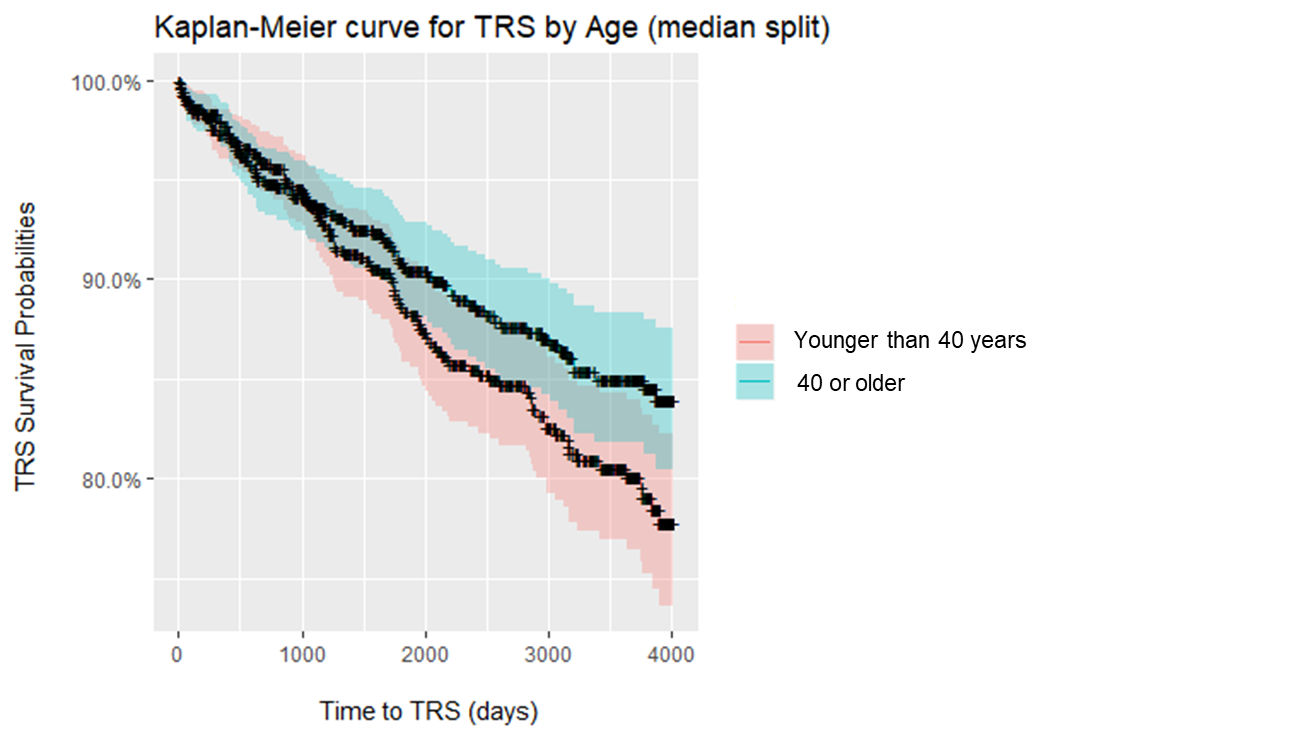

Supplement: S7 Fig — (DOCX) [file pone.0274864.s013.docx]
