# Supplementary material for: A predictor model of treatment resistance in schizophrenia using data from electronic health records
Source: PLoS One. 2022 Sep 19;17(9):e0274864. doi: 10.1371/journal.pone.0274864 (PMC9484642; doi:10.1371/journal.pone.0274864)

**Supplementary Figure 8**: **Kaplan-Meier curve of the survival probabilities for treatment resistant schizophrenia (TRS) by number of inpatient days before the prescription of the first antipsychotic from 01/01/2007 (median split of 4 inpatient days)**


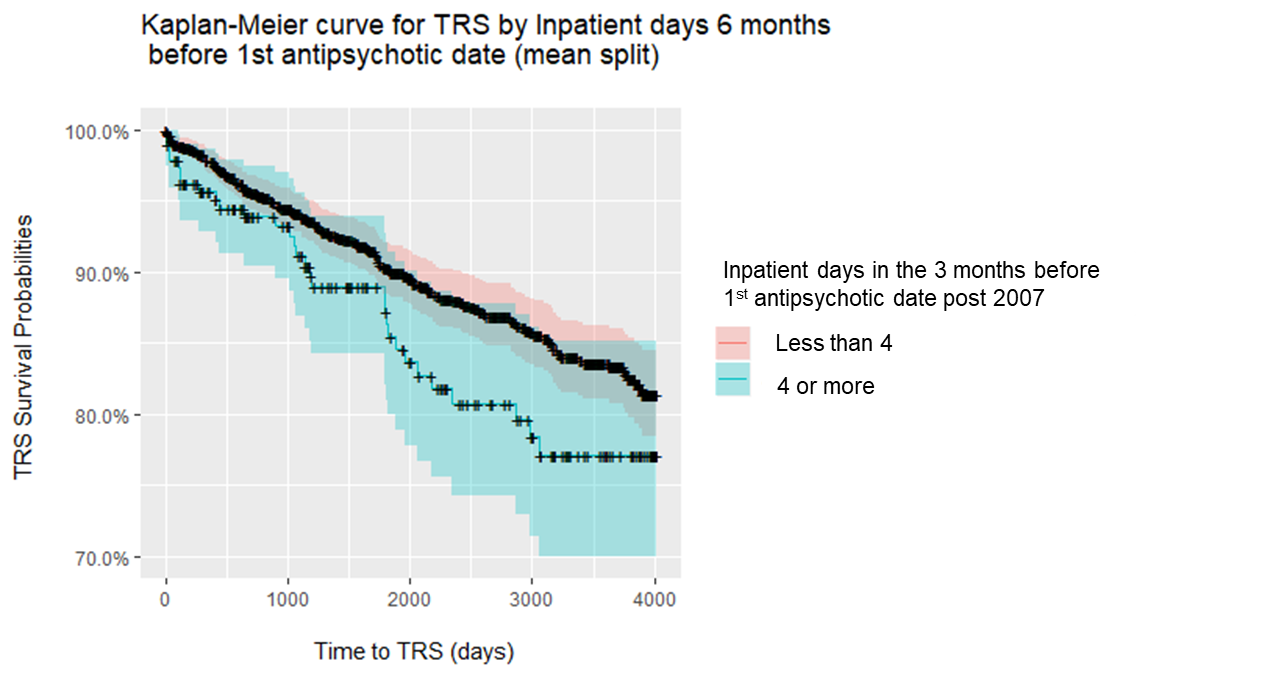

Supplement: S8 Fig — (DOCX) [file pone.0274864.s014.docx]
