# Supplementary material for: A predictor model of treatment resistance in schizophrenia using data from electronic health records
Source: PLoS One. 2022 Sep 19;17(9):e0274864. doi: 10.1371/journal.pone.0274864 (PMC9484642; doi:10.1371/journal.pone.0274864)

**Supplementary Figure 10**: **Kaplan-Meier curve of the survival probabilities for treatment resistant schizophrenia (TRS) by number of face-to-face clinical contacts in the three months before the prescription of the first antipsychotic from 01/01/2007 (median split of 3 clinical contacts)**


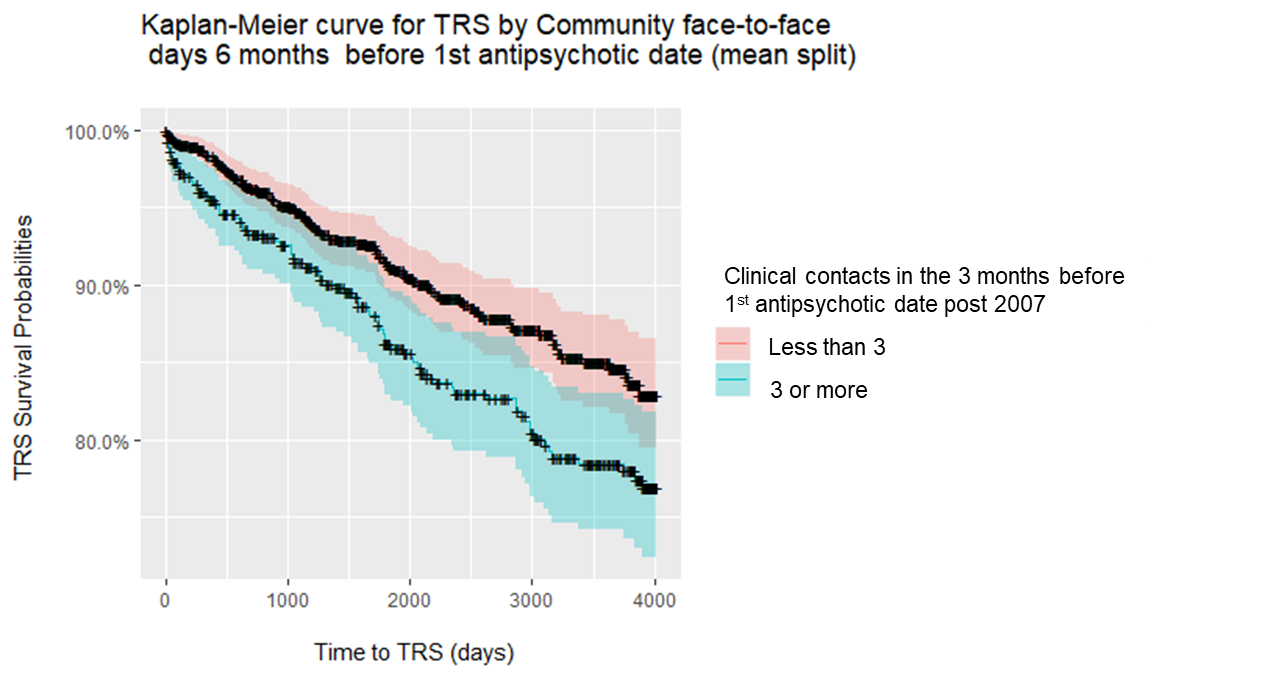

Supplement: S10 Fig — (DOCX) [file pone.0274864.s016.docx]
