# Supplementary material for: A predictor model of treatment resistance in schizophrenia using data from electronic health records
Source: PLoS One. 2022 Sep 19;17(9):e0274864. doi: 10.1371/journal.pone.0274864 (PMC9484642; doi:10.1371/journal.pone.0274864)
Supplement: S1 Appendix — (DOCX) [file pone.0274864.s017.docx]

The nomogram allows us to compute the normalized prognostic index (PI) for a new patient. The PI is a single-number summary of the combined effects of a patient’s risk factors and is a common method of describing the risk for an individual. In other words, the PI is a linear combination of the risk factors, with the estimated regression coefficients as weights. The exponentiated PI gives the relative risk of each patient in comparison with a baseline patient. The PI is normalized by subtracting the mean PI. To predict individual normalized PI for TRS for a new patient using the nomogram, one can take the example of a 30-year-old patient, with Schizophrenia, having 34 inpatient days recorded at 3 months before the 1st antipsychotic (AP) date, 50 inpatient days recorded 3 months after 1st AP date, 15 community face-to-face days recorded 3 months before 1st AP date, having a minor problem requiring no action and having mood disorders as comorbidity, has a total point score of 78 + 33 + 28 + 6 + 18 + 10 + 0 = 173. This corresponds to a normalized prognostic index of 0.57 (linear predictor line) for TRS, meaning that the patient has a probability to become TRS at 1 year falling in the range 4.14%-7.66%, at 2 years falling in the range 7.62%-13.89%, at 5 years in the range 16.45%-28.74% and at 10 years in the range 26.57%-44.13% (see Supplementary Table 3).

A more precise way to compute the probabilities of TRS at 1, 2, 5 and 10 years for a new individual is using the following formula for absolute risk predictions at time *t*:

$1-{S_{0}\left( t \right)}^{exp(b_{1}x_{1}+b_{2}x_{2}+b_{3}x_{3}+\ldots)}$,

where $S_{0}\left( t \right)$ is the baseline survival probability at time *t* (see Table A), $x_{i}$ are the variables and $b_{i}$ are the log hazard ratios, i.e. the Cox-Lasso estimated coefficients (see Table B).

For example, for the same individual as above, the probability of developing TRS at 1 year will be precisely:

$$1-0{.9624709}^{\exp\left( -0.01009\times30-0.30132\times0+0.00741\times34+0.00110\times50+0.01044\times15+0.08912\times1-0.11980\times1 \right)}=0.0426476=4.26\%$$

**Table A**: Baseline survival probability at 1, 2, 5 and 10 years.

| $\boldsymbol{t}$ **(years)** | $\boldsymbol{S}_{\boldsymbol{0}}\left( \boldsymbol{t} \right)$ |
| --- | --- |
| 1 | 0.9624709 |
| 2 | 0.9307469 |
| 5 | 0.8499845 |
| 10 | 0.7563057 |

**Table B**: Lasso-Cox coefficients

| **Selected variables** | | **Coefficients (log hazard ratios)** | | |
| --- | --- | --- | --- | --- |
| $\boldsymbol{x}_{\boldsymbol{1}}$ | Age | | $\boldsymbol{b}_{\boldsymbol{1}}$ | -0.010086 |
| $\boldsymbol{x}_{\boldsymbol{2}}$ | SCZ spectrum diagnosis (1=other prolonged psychosis, 0=SCZ/schizoaffective/undetermined) | | $\boldsymbol{b}_{\boldsymbol{2}}$ | -0.301323 |
| $\boldsymbol{x}_{\boldsymbol{3}}$ | Inpatient days pre index | | $\boldsymbol{b}_{\boldsymbol{3}}$ | 0.007413 |
| $\boldsymbol{x}_{\boldsymbol{4}}$ | Inpatient days post index | | $\boldsymbol{b}_{\boldsymbol{4}}$ | 0.001103 |
| $\boldsymbol{x}_{\boldsymbol{5}}$ | Community face-to-face days pre index | | $\boldsymbol{b}_{\boldsymbol{5}}$ | 0.010438 |
| $\boldsymbol{x}_{\boldsymbol{6}}$ | HONOS Cognitive problems (1=minor problem requiring no action, 0=no problem/severe problem) | | $\boldsymbol{b}_{\boldsymbol{6}}$ | 0.089123 |
| $\boldsymbol{x}_{\boldsymbol{7}}$ | Comorbidity (1=mood disorder, 0=no mood disorder) | | $\boldsymbol{b}_{\boldsymbol{7}}$ | -0.119803 |
